# The importance of inflammation control for the treatment of chronic diabetic wounds

**DOI:** 10.1111/iwj.14048

**Published:** 2022-12-23

**Authors:** Anna L. Worsley, Dennis H. Lui, Winnie Ntow‐Boahene, Wenhui Song, Liam Good, Janice Tsui

**Affiliations:** ^1^ Royal Veterinary College Department of Pathobiology and Population Sciences London UK; ^2^ UCL Centre for Biomaterials in Surgical Reconstruction and Regeneration, Department of Surgical Biotechnology, UCL Division of Surgery and Interventional Science University College London London UK

**Keywords:** chronic wounds, diabetes, diabetic ulcers, inflammation, wound healing

## Abstract

Diabetic chronic wounds cause massive levels of patient suffering and economic problems worldwide. The state of chronic inflammation arises in response to a complex combination of diabetes mellitus‐related pathophysiologies. Advanced treatment options are available; however, many wounds still fail to heal, exacerbating morbidity and mortality. This review describes the chronic inflammation pathophysiologies in diabetic ulcers and treatment options that may help address this dysfunction either directly or indirectly. We suggest that treatments to reduce inflammation within these complex wounds may help trigger healing.

## 1 INTRODUCTION

Diabetes mellitus is a chronic metabolic condition of insulin resistance, reduced insulin production, and chronically elevated blood glucose levels. The global prevalence of diabetes has trebled in the last two decades, particularly in countries with developing economies, and is predicted to affect 1 in 10 people worldwide by 2045.^1^ Diabetic foot ulceration is one of the major complications of diabetes. It is a serious, highly morbid condition, which has been shown to be independently associated with increased mortality.^2^ Patients with diabetes mellitus are especially prone to developing foot ulceration due to peripheral neuropathy, which leads to biomechanical changes to the foot and loss of protective reflexes and sensation to injury.^3^, ^4^, ^5^ The presence of peripheral vascular disease and a predilection to polymicrobial infection are also contributing factors. As many as one in three patients with diabetes mellitus will develop a diabetic foot ulcer during their lifetime.^3^, ^4^, ^6^, ^7^

**FIGURE 1:**
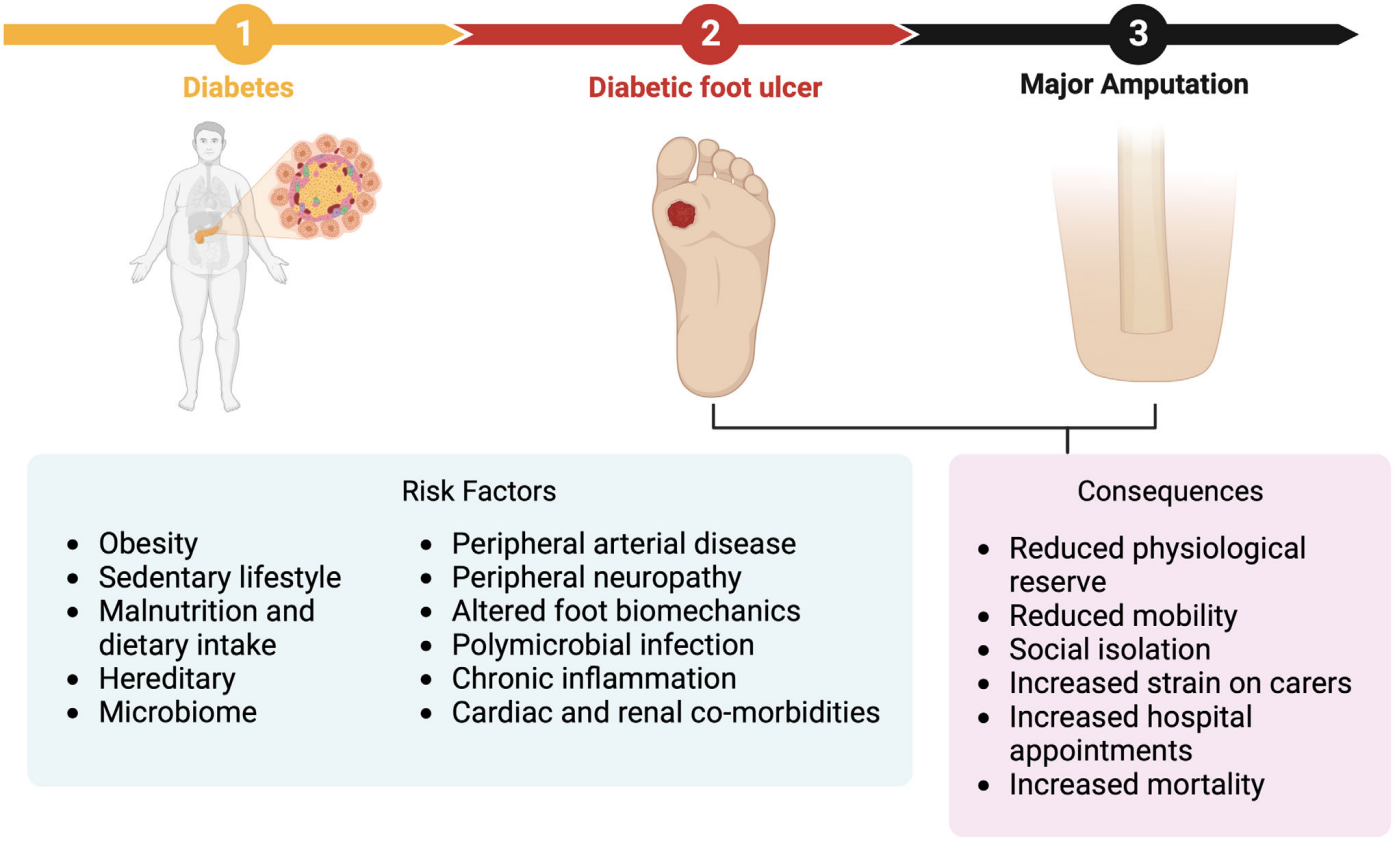
Progression, associated risk factors, and subsequent health risks of diabetic foot ulceration. Created with BioRender.com

The prognosis for patients suffering from diabetic foot ulceration is bleak. Thirty three percent of diabetic ulcers do not heal and remain as chronic wounds.^8^ Of those ulcers that do achieve ‘healing’, 65% will experience re‐ulceration at 3 years, exemplifying the chronic, relapsing, and remitting nature of this condition.^3^ Approximately 20% of moderate to severe diabetic foot ulcers lead to some form of amputation, and patients with diabetes are up to 25 times more likely to lose their leg than those not suffering from the condition.^3^ In 2005, the International Diabetes Federation estimated that one lower limb was lost every 30 seconds due to diabetes worldwide.^9^ Five‐year survival after diabetes‐related major amputation was estimated to be 47%, dropping to 17% for patients on dialysis.^10^ These survival rates are comparable to, or worse than, other severe diseases, including heart failure, myocardial infarction, stroke, and some cancers.^11^, ^12^ A large, population‐based study found that over a follow‐up period of 10 years, patients with a history of diabetic foot ulceration were twice as likely to die than those without diabetes, consistent with results found by other studies.^12^, ^13^, ^14^ The presence of foot ulceration in diabetic patients is associated with poor quality of life, low physical functioning, increased risk of depression, and anxiety, and is an independent risk factor for mortality.^2^, ^15^ The multitude of knock‐on effects an ulcer may have on a patient's health must be considered by health care professionals and carers. (Figure 1).

The burden of foot ulceration on health care systems, carers, as well as to society in terms of health economics cannot be overstated. Where diabetes‐related care was estimated to cost 176 billion USD annually, up to one third of this was attributed to lower extremity care.^3^, ^15^ The presence of diabetic foot ulceration was associated with a seven‐fold increase in requiring hospital admission, as infection and gangrene are common complications once an ulcer has formed.^16^ The costs of hospital care, outpatient clinic care, informal care, sickness absence, and care after amputation amount to tens of billions worldwide.^11^, ^17^, ^18^, ^19^, ^20^, ^21^ Worryingly, these costs are set to continue to increase as both the prevalence of diabetes and life expectancy with diabetes are rising.

**FIGURE 2:**
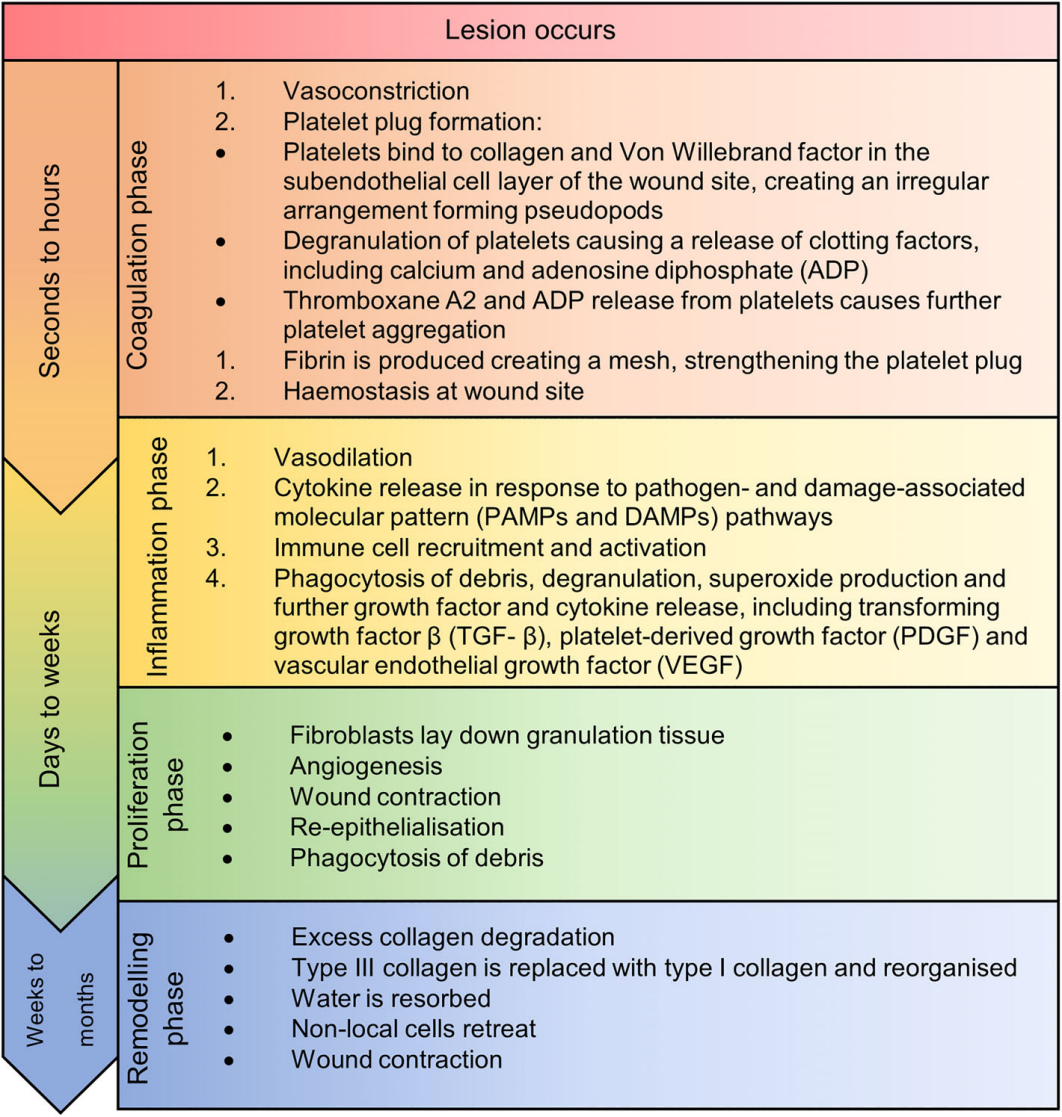
An overview of the principal stages of normal wound healing^127^, ^128^, ^129^, ^130^, ^131^, ^132^

The mammalian body has evolved a carefully orchestrated series of defences to skin wounding, which include innate defences and acquired responses. These defences always include initial inflammation, cell proliferation, differentiation and migration, angiogenesis, extracellular matrix proliferation, and remodelling. Chronic inflammation is a key feature of both diabetes mellitus and wound chronicity. Though other inflammatory conditions can lead to wound chronicity (i.e., scleroderma), diabetic patients are at particular risk due to repeated injury due to peripheral neuropathy and the loss of protective reflexes and the polymicrobial bioburden in their wounds, particularly in foot ulcers. This chronic inflammatory state in diabetes mellitus disrupts the normal responses to wounding at a systemic and local level, leading to wounds that are unable to progress through the normal wound healing stages (Figure 2).^8^ Moreover, patients with a chronic inflammatory state are less able to mount an appropriate systemic and local response to the microbial bioburden often present in wounds. This ‘burnt‐out’ innate immunity of diabetic patients, combined with the propensity for poly‐microbial wound infection in diabetic foot ulceration, are the factors, which drive the manifestation of overwhelming bacterial sepsis from diabetic foot infections, which can be life‐threatening. It is important to understand this inflammatory dysfunction in order to develop strategies to target chronic inflammation and improve wound healing without further jeopardising innate immunity to invading pathogens. This review summarises current knowledge concerning the role of chronic inflammation in chronic diabetic wounds, including diabetic foot ulcers, and the treatment options that may help address this dysfunction either directly or indirectly.

## 2 PATHOPHYSIOLOGY

### 2.1 Reduced initial inflammatory response

**FIGURE 3:**
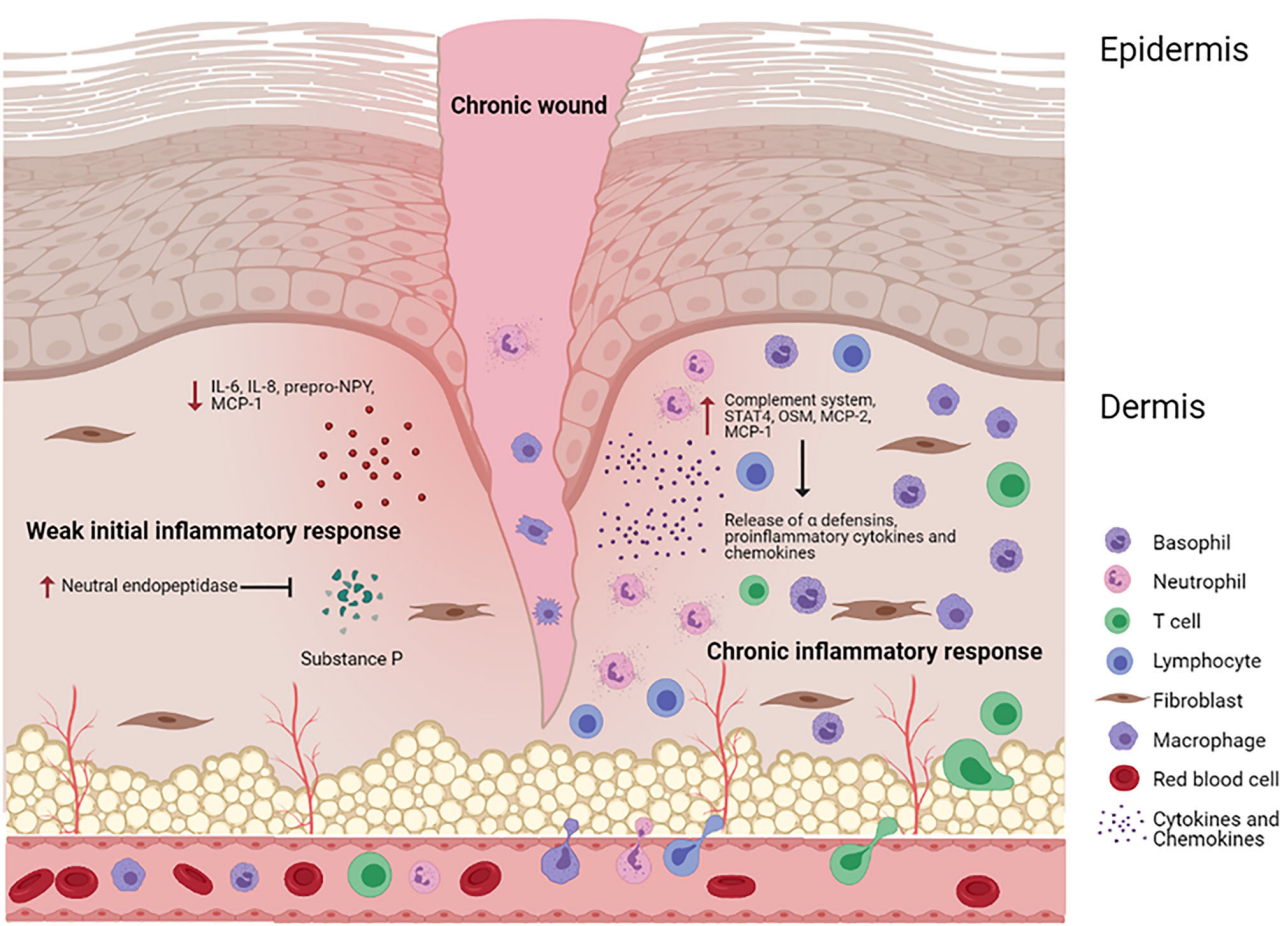
Pathophysiology of diabetic chronic wounds. The condition illustrated arises through two stages. In the first stage there is a weak initial inflammatory response which is then followed by the chronic inflammatory response. During the weak initial inflammatory response, low levels of cytokines (IL‐6, IL‐8) CCR2 and prepro‐NPY are released within the wound environment. The levels of neutral endopeptidase, an enzyme which degrades substance P, also increases in the skin. In the second stage, there is reduced keratinocyte, fibroblast and endothelial cell stimulation. In contrast, the chronic inflammatory response is characterised by an accumulation of leukocytes (macrophages, neutrophils, basophils and T cells) due to an overexpression of the complement system, STAT4, OSM and chemoattractants MCP‐2 and MCP‐1. In addition, the dysregulation of SELPLG also reduces the clearance of these leukocytes from the wound site

Relative to acute wounds, diabetic chronic wounds display weak early‐stage inflammatory responses (Figure 3), which may underlie the development of the chronic inflammatory phenotype of diabetic ulcers. At wounding, IL‐6, IL‐8, their receptors, the C‐C chemokine receptor type 2 (CCR2) for macrophage chemoattractant protein‐1 and prepro‐NPY, the precursor of neuropeptide Y (NPY), are present at much lower levels compared with non‐diabetic environments .^22^, ^23^, ^24^ This is also mirrored in single‐cell transcriptomic and pathways analysis showing that systemic NK and T cells in diabetic patients exhibit inhibition of IL‐6, IL‐8, and CD28 signalling pathways.^25^ There is also an increase of neutral endopeptidase in the skin around patients with diabetic chronic wounds. Neutral endopeptidase degrades substance P as part of its regulation, suggesting a reduction in substance P.^26^ The neuropeptide substance P stimulates keratinocyte, fibroblast, and endothelial cell pro‐inflammatory responses.^26^ These differences in the diabetic wound environment suggest a reduced initial inflammatory response compared with non‐diabetic acute wounds.^22^, ^23^, ^24^, ^26^

### 2.2 The chronic inflammatory phenotype

Immune cell infiltration, accumulation, and pro‐inflammatory cell polarisations are promoted in the diabetic chronic wound environment, contributing to the chronic inflammatory phenotype (Figure 3).^27^ The expression levels of the complement system, signal transducer and activator of transcription factor (STAT) 4, oncostatin M (OSM), OSM receptor subunit β (OSMRβ), macrophage inflammatory protein‐2, and macrophage chemoattractant protein‐1 expression have all been found to be increased in the diabetic ulcer environment.^24^, ^28^ This contributes to the accumulation of polymorphonuclear neutrophils and macrophages. Macrophages become hyperpolarised and are predominantly granulocytic (Gr) ‐1+, CD11b+, and CD14+ macrophages.^29^ Their clearance is reduced in association with the dysregulation of the cell membrane protein selectin P ligand (SELPLG), causing a build‐up of their population and associated secreted pro‐inflammatory molecules.^30^ α‐defensins are also upregulated in hyperglycaemic conditions, promoting IL‐8 expression and enhancing the recruitment of neutrophils, basophils, and T cells.^31^, ^32^ This influx and accumulation of immune system cells is followed by the release of associated pro‐inflammatory cytokines and chemokines, worsening the chronic inflammatory phenotype. Infection worsens the situation by promoting immune responses as well as complicating ischaemia and neuropathy.^33^ Excretions from *S. aureus* biofilms, the dominating bacteria species in diabetic chronic wounds, were also found to directly contribute to the chronic inflammatory phenotype by causing pro‐inflammatory gene expression in epithelial keratinocytes.^34^

#### 2.2.1 Senescence‐associated secretory phenotype

Cellular senescence describes the process in which cells cease dividing and undergo phenotypic changes. As with many tissues in patients with diabetes, diabetic ulcers have an increased number of senescent cells displaying a pro‐inflammatory senescence‐associated secretory phenotype (SASP). This phenotype develops as a consequence of increased oxidative stress‐related RNA damage, DNA damage, and telomere shortening.^35^, ^36^ Experimental models of diabetic mice revealed a rapid accumulation of senescence cells of various cell types in wounded skin.^37^ The majority of SASP cells are macrophages, which are hypothesised to be stalled between the transition from pro‐inflammatory M1 and pro‐resolution M2 phenotypes. Fibroblasts also show a strong senescent phenotype, adding to poor healing outcomes.^38^ This is potentially in response to hyperglycaemia and increased local inflammation.^35^, ^39^ A SASP increases the release of pro‐inflammatory cytokines and chemokines, promoting a chronic inflammatory environment. CXC chemokine receptor 2 (CXCR2), the IL‐8 receptor, as well as chemokines chemokine (C‐X‐C motif) ligand 1 (CXCL) 1 and CXCL2 are highly expressed in senescent cells, potentially playing a role in the chronic inflammatory pathophysiology through immune cell recruitment.^31^, ^32^, ^38^ Inhibition of CXCR2 has been shown to protect CXCL2 activity, dampen neutrophil infiltration, and reduce cellular senescence, promoting wound closure in diabetic ulcers. This suggests CXCR2 and IL‐8 play important roles in diabetic chronic wound pathophysiology.^38^ SASPs may also contribute to diabetic ulcer formation as part of metaflammation. Monocyte chemoattractant protein‐1 (MCP‐1) is a pro‐inflammatory cytokine expressed in SASP cells. MCP‐1 is upregulated in diabetic rat models and has been found to be associated with patient susceptibility to diabetic ulcer formation.^40^

#### 2.2.2 Neutrophils

In the diabetic environment, neutrophils display increased superoxide production and protein kinase C activity.^41^ They also have elevated basal calcium levels, overproduce peptidyl arginine deiminase type IV (PAD4), a calcium‐dependent enzyme, and overproduce neutrophil extracellular traps (NETs), impeding wound healing via increased NETosis.^42^ Despite this increased NETosis, it was found that in diabetic mouse wounds infected with *S. aureus* neutrophil apoptosis was reduced, neutrophil clearance was reduced and neutrophil TNF‐α production increased.^43^ Elevated saturated free fatty acids also promote neutrophil survival and reduce macrophage phagocytosis in association with prostaglandin production.^44^ These processes cause the accumulation of neutrophils in diabetic ulcers, adding to the increased inflammation and reduced wound healing progression.

#### 2.2.3 Macrophages

The persistence of pro‐inflammatory M1 macrophages within wounds has been hypothesised to be a key contributor to diabetic chronic wound pathology. However, newer transcriptomic data has identified that, at least in the wound, the M1 phenotype may also be associated with improved diabetic foot ulcer healing.^25^ Driven by hyperglycaemia and hypoxia, the pro‐inflammatory phenotype is characterised by an increased release of inflammatory cytokines such as TNF‐α and IL‐1.^45^ TNF‐α stimulates the histone acetyltransferase Males absent on the first (MOF) in macrophages and that this is increased in the diabetic environment. MOF adds to the activation of TNF‐α signalling and promotes NFκB–mediated gene transcription via H4K16 acetylation in wound macrophages, impeding wound healing processes.^45^ In an obese mouse model, inhibiting TNF‐α signalling using neutralising monoclonal antibodies inactivated macrophages, reduced circulating monocyte populations and reduced inflammatory cytokine levels. This induced wound closure, suggesting TNF‐α signalling is another major contributing factor to diabetic ulcer development.^46^

Sustained elevated expression of IL‐1β by the nodulation (NOD)‐, Leucine‐rich repeat (LRR)‐, and pyrin domain‐containing protein 3 (NLRP3) inflammasome further impedes macrophage transition to the pro‐healing M2 phenotype by downregulating peroxisome proliferator‐activated receptor (PPAR) ‐γ, a regulator of glucose and lipid metabolism.^47^ Insulin treatment may reduce these effects. In vivo studies indicated that insulin promotes anti‐inflammatory phenotype transitions by upregulating PPAR‐γ. Furthermore, insulin activated Akt‐Rac‐1 (activated cell division control protein 42 kinase‐ras‐related C3 botulinum toxin substrate‐1) signalling, a regulatory pathway for glucose uptake that is downregulated in diabetic ulcers, inhibiting hyperglycaemia‐induced p38, NF‐κB, and STAT1 transcriptional activity activation.^48^ The neuropeptide neurotensin has also been found to be involved in stimulating the migratory and inflammatory response of macrophages in hyperglycaemic environments, indicating the role of the neuroendocrine system on diabetic wound healing.^49^

As mentioned above, the phagocytic and efferocytotic activity of macrophages is reduced in diabetes, increasing the population of dysfunctional cells in the diabetic chronic wound environment. The phagocytic activity of macrophages for apoptotic neutrophils has been found to be significantly reduced in diabetic ulcers.^50^ Phagocytosis‐related genes for CD36 and Class B scavenger type 1 receptors are downregulated, pro‐apoptotic factors are downregulated, and pro‐apoptotic factors are upregulated in the pro‐inflammatory phenotype.^50^ Macrophage efferocytosis impairment increases the burden of apoptotic cells at the wound site, causing the stimulation of pro‐inflammatory and the attenuation of anti‐inflammatory cytokine responses.^51^

#### 2.2.4 γδ T lymphocytes

Diabetic chronic wounds have lower numbers of skin‐resident γδ T lymphocytes, of which have reduced expression levels of FGF‐7, FGF‐10, and insulin‐like growth factor (IGF) ‐1 compared with acute healing models.^52^ These growth factors are more pro‐healing than pro‐inflammatory, and in general, T lymphocytes have a more anti‐inflammatory phenotype.^53^, ^54^, ^55^ Reduced population and activity of skin‐resident γδ T lymphocytes further promotes inflammation.

#### 2.2.5 Other dysfunctional signalling molecules and receptors

Multiple areas of dysfunction cause the pro‐inflammatory phenotype found in diabetic ulcers. The dysregulation of upstream receptors that initiate inflammatory cascades, such as the toll‐like receptor (TLR) family, plays an important role. TLRs 2, 4, 7, and 9 are significantly upregulated in diabetic chronic wounds.^6^, ^8^, ^56^, ^57^, ^58^, ^59^ Antagonising TLR‐4 systemically in vivo or using knockout TLR‐4 in vivo models has been found to improve diabetic wound healing, and reduce pro‐inflammatory phenotypes.^8^, ^59^ In contrast, clinical studies of diabetic foot ulcers in patients with Indian heritage found reduced levels of TLR‐4 signalling in diabetic foot ulcers with specific TLR‐4 SNP genotypes, displaying potential variation in how TLR‐4 is dysfunctional.^60^ The increase in TLR expression in diabetic ulcers reduces healing, increases MyD88 signalling and increases the expression and activation of NF‐κB, interferon and inflammatory cytokines and chemokines, such as IL‐6, TNF‐α, S100A8, Il‐8, and Il‐1β.^6^, ^8^, ^56^, ^57^, ^58^ Many of these signalling molecules go on to promote further pro‐inflammatory cytokine release as part of positive feedback loops, worsening the inflammatory phenotype.

Many other mechanisms further downstream contribute to the dysfunctional healing seen in diabetic ulcers. For example, unregulated iron levels lead to reactive oxygen species (ROS) production, increased oxidative stress, and macrophage polarisation.^38^, ^61^, ^62^ Granzyme B is a serine protease, which positively regulates apoptosis in normal wound healing. Expressed on immune system cells, it accumulates in the extracellular matrix (ECM) in the diabetic ulcer environment. It cleaves essential wound healing proteins such as fibronectin, preventing wound healing.^63^ Sirtuin 6 is a sirtuin family protein involved in regulating many pathophysiological processes, including inflammation, glycolysis and DNA repair.^64^ Sirtuin 6 deficiency in diabetic chronic wounds further exacerbates the pro‐inflammatory phenotype of diabetic chronic wounds by increasing NF‐κB activation, oxidative stress, and decreasing angiogenesis.^64^

Chronic hyperglycaemia has been shown to upregulate the signalling molecule suppressor of cytokine signalling 3 (SOCS3), a protein usually involved in the suppression of inflammation.^65^ Surprisingly, in the diabetic chronic wound environment, this exacerbates wound inflammation. This is in association with increased expression of chemokine macrophage inflammatory protein 2 (MIP‐2), increased expression of inflammatory enzymes cyclooxygenase (COX)‐2, inducible nitric oxide synthase (iNOS), and increased levels of TGF‐ β in epithelial cells.^66^ COX‐1 and ‐2 expression and activity have also been found to be dysregulated using diabetic obese mouse wound models, with COX‐1 coupled prostaglandin directly contributing to impaired diabetic wound healing.^67^

Endothelial overexpressed lipopolysaccharide‐associated factor 1 (EOLA1) is a recently discovered regulator of inflammation, which is downregulated in diabetic chronic wounds.^68^ EOLA1 is expressed in leukocytes and endothelial cells. It has been found to be involved in cell growth promotion, apoptosis inhibition, and the downregulation of inflammatory cytokine secretion, including IL‐6 and intercellular adhesion molecule‐1 (ICAM‐1).^68^ Therefore, its downregulation may play an important role in the chronic inflammatory phenotype of diabetic ulcers. In contrast, it is upregulated after LPS stimulation, suggesting a potential mechanism involved in reducing the diabetic ulcer's ability to fight infection.

## 3 CURRENT TREATMENTS THAT REDUCE INFLAMMATION

### 3.1 Commonly used treatments and management techniques

Many of the management principles and treatment options used to treat diabetic foot ulceration have indirect anti‐inflammatory effects. Diabetic chronic wounds remain open for extended periods of time, often months, due to dysfunctional immune responses. When considering that open diabetic wounds also encourage polymicrobial colonisation, unsurprisingly the rate of wound infection and deeper infection (including osteomyelitis) is high, with approximately 58% of cases being infected.^69^, ^70^, ^71^, ^72^ Infection, where present, must be controlled as a first priority. Localised infection increases inflammation, exacerbates the dysfunctional immune response, increases pain, and promotes morbidity.^3^, ^51^, ^73^ Treatment for infection in these wounds is achieved with (1) prompt surgical debridement and drainage of any collections, if necessary, and (2) empirical antibiotics followed by sensitivity‐guided antibiotics after identification of bacterial strains is complete from tissue sampling.^74^ Surgical debridement is performed to clean the wound, removing necrotic, non‐viable, or infected tissue as well as building up immune cells and cytokines in the exudate and slough, thereby reducing their pro‐inflammatory output.^75^ Dressings can also contain antiseptic agents, such as silver, iodine, and polyhexamethylene biguanide (PHMB) to help stabilise the current infection and prevent further infection.^76^, ^77^ As well as benefiting wound healing, incorporating antimicrobials into dressings can improve pain, and reduce the risk of further complications, such as sepsis.^70^ Routine wound care, including regular irrigation and prompt removal of excessively soiled dressings, may also reduce excessive inflammation.

Negative pressure wound therapy (NPWT) is a treatment, which may be applied after debridement and infection control to help promote wound approximation and healing.^74^ It achieves this effect by removing excess wound exudate, inducing wound contraction, and promoting angiogenesis as well as wound granulation through mechanical stimulation.^78^ NPWT may also reduce inflammation, however, reports are mixed. Wang et al reported that negative pressure therapy suppresses inflammation via down‐regulating the MAPK‐JNK signalling pathway in diabetic ulcers.^79^ Ludwig‐Slomczynska et al found that NPWT for diabetic ulcers caused epigenetic changes that lead to the inhibition of complement system activation.^80^ In contrast, Pawar et al recorded increased CD68 cell densities in response to NPWT for periprosthetic tissue treatment, indicating increased inflammation.^81^ Furthermore, Norbury et al found that NPWT helped overcome immunoparalysis in the swine model of ischemia/reperfusion injury coupled with sepsis, specifically by increasing lymphocyte populations and increasing macrophage reactive oxygen species production.^82^ The International Working Group on the Diabetic Foot (IWGDF) performed a systematic review of literature on the use of interventions to enhance healing in chronic diabetic foot ulcers, including the use of NPWT.^83^ The working group recommended that NPWT should be considered to reduce wound size in post‐surgical wounds, and that it should be avoided in non‐surgical wounds. Their published recommendations take into account the variability of studies, the lack of blinding and the controls in studies, and potential for bias in the current literature.

### 3.2 Less common and future treatments

#### 3.2.1 Hyperbaric oxygen therapy

Hyperbaric oxygen (HBO_2_) as a therapy has been studied in animal models and as a treatment for human injury and wound healing for more than four decades.^84^, ^85^ The treatment involves controlled exposure of the patient to high atmospheric pressure (between 2 and 3 atm), with 100% oxygen content in a compression chamber for a set amount of time (1–2 hours). The net effect of this therapy is to temporarily increase oxygen tension in the wound, followed by a decrease back to hypoxic conditions, and cycling between these two states appears to improve wound healing. Hyperbaric oxygen therapy reduces expression of inflammatory cytokines (including Interleukin‐1 and Interleukin‐2), increases angiogenesis, improves collagen formation, promotes fibroblast migration, reduces the metalloproteinase expression, and promotes both antibiotic and leucocyte function against microbes.^85^, ^86^, ^87^, ^88^ Studies in various animal models have demonstrated that wounds exposed to hyperbaric oxygen show increased granulation and accelerated wound contraction, as well as ameliorating inflammatory processes in other conditions.^86^, ^89^ However, the treatment does have risks, including barotrauma to the ears, seizures from acute central nervous system oxygen toxicity, and reversible myopia. A Cochrane review and meta‐analysis of HBO_2_ therapy for chronic wounds found moderate evidence that the treatment improved the chance of healing at 1 year in diabetic foot ulcers, but did not reduce the risk of amputation.^84^ The provision of hyperbaric oxygen therapy requires specialist facilities, which are relatively more available in North America, but less common elsewhere in the world, limiting widespread adoption. Current guidance from the National Institute of Health and Care Excellence (NICE) does not recommend the use of hyperbaric oxygen in chronic diabetic foot wounds except when involved in a clinical trial.^74^

#### 3.2.2 Skin replacement and grafting, and amniotic membrane treatment

Replacement of epithelial tissue with autograft, allograft, xenograft, or bioengineered tissues has been studied as a treatment for chronic diabetic foot wounds. These skin substitutes are categorised by whether they are cellular or acellular scaffolds made from skin tissues or biomaterials and are further categorised as dermal or epidermal substitutes (or both). When applied to infection‐free and vascularised wounds, these tissues may integrate with the wound and act to improve extracellular matrix deposition and composition, and increase secretion of healing cytokines and growth factors, and ultimately increase re‐epithelialisation.^90^ However, the exact effect of these treatments on chronic wound inflammation is not yet fully characterised. Numerous bioengineered skin substitutes containing fresh or cryopreserved human keratinocytes or fibroblasts have been applied to patients with diabetic foot ulceration, with positive preliminary results.^91^, ^92^, ^93^, ^94^ Unfortunately, the significant heterogeneity of skin substitute products available, heterogenous study design without blinding investigators, low study numbers, industry involvement in the majority of studies, and potential for bias all mean that at present, it is difficult to draw any firm conclusions about this potentially promising treatment.^90^, ^91^

Application of human amniotic membrane is another therapy, which may reduce inflammation in diabetic ulcers. Dehydrated or cryopreserved allogeneic human amniotic membrane is now commercially available, and its application in diabetic foot ulceration has been studied. Amniotic membrane tissue is rich in cytokines (IL‐4, IL‐6, IL‐8, and IL‐10), growth factors (including platelet derived growth factors, fibroblast growth factors, transforming growth factor α, epidermal growth factor, and granulocyte colony‐stimulating factor), native stem cells, and tissue inhibitors of metalloproteinases (TIMPs).^95^, ^96^, ^97^, ^98^ Amniotic membrane has been shown to reduce Th1 and Th2 cell cytokine synthesis and also lacks human leukocyte antigen (HLA) antigens, which make it less immunogenic compared with other skin substitutes.^97^, ^99^ Clinical studies examining human amniotic membrane allograft in chronic diabetic foot ulcers have shown improved wound healing in diabetic ulcers.^90^, ^97^, ^98^, ^100^ However, these trials were small, non‐blinded, and had industry involvement. Use of these products has been limited partly due to the prohibitive cost of each treatment cycle and the lack of clear long‐term evidence. At present, NICE guidelines suggest skin replacements can be considered only in diabetic foot ulcers, which have failed to progress, at the recommendation of a multidisciplinary foot care service.^74^ Further well‐designed studies are required to examine the physiological effects and clinical effects, including long‐term effects, of skin substitutes and grafting in chronic diabetic wounds.

#### 3.2.3 Growth factors

Delivering growth factors to the wound bed may help reduce inflammation as well as general wound healing promotion. Growth factors can be delivered directly via injection or by incorporation into biomaterials, or indirectly via stem cells, exosomes, platelet‐rich‐plasma, or allographs.^75^, ^101^ Growth factors used directly for diabetic foot ulcer treatments described in the literature include TGF‐β, FGF, VEGF, PDGF, and EGF, the latter two being the only ones to reach clinical evaluation.^102^ PDGF and EGF have anti‐inflammatory properties when used in skin disorders.^103^, ^104^ In pre‐clinical and clinical studies, EGF has been shown to improve healing with the promotion of granulation and fibroblast proliferation.^101^, ^105^, ^106^, ^107^ Regranex is a gel containing PDGF; it is the only growth factor treatment approved by the United States food and drug administration, however, it is not recommended by The National Institute for Health and Care Excellence (NICE).^74^ This is mainly in response to the formulation, dosing, safety, and efficacy issues.^53^ One example raising safety concerns includes a retrospective observational study connecting Regranex to an increased risk of malignancies. Despite this study being unpublished and requiring further evidence, its use in high risk cancer patients is restricted in the United Kingdom as a consequence.^108^ The clinical evidence of efficacy for Regranex has been seen as weak, and the product is not widely used.^109^

#### 3.2.4 Stem cell therapy

Stem cell therapy can be used to deliver active molecules and complexes to the wound bed. Its recommendation is not directly mentioned in NICE guidelines, however, it is sometimes used as a last attempt to avoid amputation in patients with no other revascularisation options.^110^ Depending on the stem cells used, they can differentiate into various cell types that help promote healing and reduce inflammation. Stem cells secrete pro‐healing cytokines, chemokines, and growth factors, including VEGFs and IGF‐1. This aids immunomodulation, cell recruitment, ECM remodeling, angiogenesis, and neuroregeneration.^110^, ^111^, ^112^ The most common stem cells used in the literature are bone marrow‐derived mesenchymal stem cells, consisting of 50% and 53% of pre‐clinical and clinical studies, respectively. Others include human umbilical cord mesenchymal stem cells and adipose tissue‐derived mesenchymal stem cells. Induced pluripotent stem cells (iPS) have not yet entered the literature for this use.^110^ Though, recently, Gorecka et al described the use of smooth muscle cells derived from iPS cells, showing the promotion of angiogenesis and accelerated healing in vivo when incorporated into collagen scaffolds.^113^ Preclinical and clinical evidence suggests that stem cells offer an effective treatment route for diabetic ulcers.^110^ Stem cell administration techniques are varied; they include local injection, the most common method, and topically as part of a free liquid, hydrogel, or solid scaffold. More research is needed to distinguish the optimal type and delivery technique, as well as the eligibility of patients and their wounds.^110^, ^112^

The use of stem cell‐derived exosomes entered the literature more recently, offering a potential route for supplying the benefits of stem cells with reduced risks and costs. In preclinical studies, stem cell‐derived exosomes promoted anti‐inflammatory cell phenotypes, cell migration, and angiogenesis.^114^, ^115^, ^116^, ^117^ This was improved when the exosomes were from LPS‐preconditioned mesenchymal stem cells compared with non‐preconditioned cells.^118^ The associated ablation of inflammation and induced wound healing was found to be through lethal‐7b miRNA shuttling associated with reduced TLR‐4 signalling, promoting M2 macrophage phenotype transitions. This adds to the evidence that TLR‐4 signalling dysfunction might play a major role in upstream diabetic wound healing prevention.

#### 3.2.5 Receptor targets

Targeting cell signalling pathways via receptor targets offers a relatively new approach in the attempt to induce healing in diabetic ulcers by reducing inflammation directly. There are many receptors identified in the literature, which have dysfunctional inflammatory signalling that could act as potential therapeutic targets for diabetic ulcers in future treatments. For example, the antagonism of members of the TLR family, such as TLR‐4, TLR‐2, TLR‐7, and TLR‐9, as well as CXCR2 and OSMRβ, some of which have been tested in vivo already.^8^, ^56^, ^57^, ^58^, ^59^, ^119^ Naltrexone is one such drug, repurposed from use as an opiate antagonist for alcohol and opioid dependence. Recently, naltrexone has been shown to promote diabetic ulcer healing in vivo by 13% to 30% via upstream receptor targeting, offering novel future treatment pathways.^119^ This may work through the inhibition of pro‐inflammatory signalling induced by TLR‐4, TLR‐7, and TLR‐9.^120^, ^121^

#### 3.2.6 Non‐specific anti‐inflammatory compounds

Numerous substances display non‐specific anti‐inflammatory activity with varying potencies. However, most are still in the preclinical stages of evaluation. Insulin, applied topically, has been found to accelerate diabetic wound healing by promoting anti‐inflammatory macrophage phenotypes.^76^, ^122^ Hyaluronic acid provides anti‐inflammatory activity in a molecular weight dependent manner with promising wound healing ability in vivo.^123^, ^124^ More naturally sourced substances, for example, honey, curcumin, oregano extract, and hydroxytyrosol, can also provide anti‐inflammatory activity.^125^, ^126^ Many are non‐specific and are still in research for their effectiveness in vivo. They are sometimes incorporated into more complex biomaterials with promising preclinical results or into simple wound dressings for a more natural approach.

## 4 CONCLUDING REMARKS

This review highlights the pivotal role of disordered inflammation in diabetic chronic wounds. Future treatments that target and break the cycle of disordered inflammation may improve treatments. Current clinical guidelines do not include specific anti‐inflammatory therapies. However, there is growing evidence that inflammation control may help overcome healing deficiencies associated with current approaches. Many potential anti‐inflammatory therapeutic options and novel research pathways are available; for example, targeting upstream receptors and the use of less‐specific anti‐inflammatory peptides. These approaches could be used alongside current treatments, providing a multi‐faceted control, which seems appropriate for a multi‐faceted condition, and a combined approach may improve patient outcomes. Further investigation of pharmacological interventions on the TLR family, systemic anti‐inflammatories including blockade of the IL‐1 axis (ie, monoclonal antibodies to IL‐1 receptor or IL‐1β), application of autologous PBMC‐derived products to wounds, and improved dressing technologies which allow optimal wound healing conditions whilst delivering topical agents, are all practical areas worthy of further investigation as future therapies.

## AUTHOR CONTRIBUTIONS

Anna Worsley, Liam Good, Wenhui Song and Janice Tsui conceptualised the manuscript. Anna Worsley and Dennis Lui wrote the manuscript. All authors read, discussed, commented and revised the manuscript.

## CONFLICT OF INTEREST

The authors declare that they have no known competing financial interests or personal relationships that could have appeared to influence the content reported in this review article.

## Data Availability

Data sharing not applicable to this article as no datasets were generated or analysed during the current study.
